# Mindfulness training during brief periods of hospitalization in multiple sclerosis (MS): beneficial alterations in fatigue and the mediating role of depression

**DOI:** 10.1186/s12883-021-02390-7

**Published:** 2021-10-08

**Authors:** Torsten Sauder, Sascha Hansen, Carina Bauswein, Roy Müller, Sonja Jaruszowic, Jana Keune, Thomas Schenk, Patrick Oschmann, Philipp M. Keune

**Affiliations:** 1 Department of Neurology, Klinikum Bayreuth GmbH, Bayreuth, Germany; 2 Department of Psychology, Ludwig-Maximilians-University of Munich, Munich, Germany; 3 Department of Physiological Psychology, Otto-Friedrich-University of Bamberg, Bamberg, Germany

**Keywords:** Multiple sclerosis, Mindfulness, Depression, Fatigue, Rumination, Acute-care hospital

## Abstract

**Objectives:**

Persons with MS (PwMS) are frequently affected by fatigue and depression. Mindfulness-based interventions may reduce these symptoms in PwMS and consequently their application has been extended to various settings. Only few efforts have been made to explore effects of short-term mindfulness training during brief periods of hospitalization. In the current study, the feasibility and potential effects of short-term mindfulness training on depression, fatigue, rumination and cognition were explored in PwMS in an acute-care hospital setting. Based on previous work, it was further examined whether the relation between trait mindfulness and fatigue prior to and following the intervention was mediated by depression and whether a mediation effect was also observable throughout the intervention.

**Methods:**

A short-term mindfulness training protocol was developed, tailored to the requirements of the acute-care setting. Subsequently, 30 PwMS were recruited sequentially and received mindfulness training during the routine clinical process (median duration in hospital: eight days, number of sessions: four). Participants completed relevant self-report measures (depression, fatigue, rumination) and a neuropsychological assessment before and after training.

**Results:**

Participants reported significantly increased trait mindfulness and decreased depression and fatigue following the intervention. Respective change scores were highly correlated so that increased trait mindfulness was associated with decreased symptoms. In the rumination domain, patients reported a tendency for an increased adaptive ability to engage in distractive behavior during arising negative mood. Other measures of trait rumination and cognition remained relatively stable. Results of the mediation analyses indicated that depression mediated the negative relationship between trait mindfulness and fatigue symptoms at pre and post assessments. With regards to the change scores, an association between mindfulness and cognitive fatigue ceased to be significant when depression was controlled, albeit in this case, the mediation effect did not reach significance.

**Conclusion:**

Results of the current study indicate that short-term mindfulness training during brief periods of hospitalization may be beneficial for PwMS. They further complement previous work by identifying depression as a potential mediator of the antagonistic relationship between mindfulness and fatigue. Based on the current exploratory study, future trials are warranted to address this mechanism of mindfulness training in more detail.

## Introduction

Multiple sclerosis (MS) is an inflammatory neurological disease in which an autoimmune response leads to demyelinization and axonal degeneration in the central nervous system. Depending on the localization of inflammatory lesions, persons with MS (PwMS) may experience various symptoms, including cognitive deficits, sensory and motor dysfunction [1–9]. Additionally, PwMS are frequently affected by depressive symptoms and fatigue. Fatigue has been described as “a subjective lack of physical and/or mental energy that is perceived by the individual or caregiver to interfere with usual or desired activity” [10]. Prevalence rates of approximately 30% for depression [11] and at least 75% for fatigue have been reported [12]. Cognitive impairments, on the other hand, occur in 40–60% of the cases [13].

In PwMS fatigue, depression and cognitive deficits are known to be strongly associated with each other [14] and adversely affect quality of life [15]. For this reason, economic and accessible treatment approaches that effectively address these symptoms are required. Mindfulness-based interventions (MBIs) represent a promising approach, with Mindfulness-Based Stress Reduction (MBSR [16] and Mindfulness-Based Cognitive Therapy (MBCT [17]) as the most prominent ones. The general goal of mindfulness trainings is to cultivate an open and non-judgmental attitude [16] that is known to be helpful in dealing with depression, rumination and fatigue [18–22]. In a recent systematic review, MBIs were shown to be superior in efficacy over specific active comparison conditions [23], i.e., conditions containing specific therapeutic mechanisms such as intensive short-term dynamic psychotherapy [24] and of similar effectiveness as evidence-based treatments in a range of psychiatric disorders (e.g., depression, anxiety, schizophrenia, eating disorders [23]).

In this context, several studies investigated the effectiveness of MBIs in PwMS [18, 25–29]. As reported in a recent meta-analysis by Simpson et al. [28], MBIs were moderately effective in reducing depression, anxiety and psychological distress. Additionally, it has been suggested that a reduction in fatigue following MBIs may be enduring and observable even several months after the intervention [25]. With regard to potential effects of MBIs on cognitive symptoms in PwMS [30, 31], the extant literature provides preliminary support that MBIs may improve cognitive abilities, albeit further methodologically robust studies appear necessary to address this issue in more detail [30]. Over the course of time, MBIs and their positive effects on depression and fatigue received considerable attention, which led to an extension of their application in PwMS. In this context, telemedicine interventions by means of an online meditation program [32, 33] and Tai Chi training combining physical exercise and mindfulness training, have been implemented [19]. These adapted mindfulness-based interventions also yielded improved symptoms of depression in PwMS.

Recently, it has been suggested that the treatment mechanisms of MBIs in PwMS ought to be examined by obtaining information about the complex interrelations of commonly examined primary outcome variables (e.g., depression, fatigue, trait mindfulness) in context of mediation analyses [34, 35]. It has been suggested that a reduction of fatigue through mindfulness training might be primarily attributable to a reduction of depressive symptoms [35]. In the latter study by Sauder et al. [35], the negative association between trait mindfulness and fatigue was shown to be mediated by depression. However, it should be noted that these results emerged in context of the limitations of a cross-sectional design focusing on persons with relapsing remitting MS (RRMS). RRMS has been shown to be associated with lower symptoms of fatigue, attributable to a lower disability status [36], compared to primary progressive MS (PPMS) and secondary progressive MS (SPMS). Moreover, it is conceivable that results previously obtained in a cross-sectional study do not necessarily generalize to an interventional study design. Therefore, it remains to be explored if these previous findings can be replicated and extended to an interventional study design including various MS subtypes.

To the best of our knowledge, to date no attempts have been made to adapt mindfulness training to an acute-care hospital setting with relatively brief periods of hospitalization. Most PwMS visit the neurological acute-care on a regular basis for monitoring purposes, treatment optimization and treatment with immune-suppressive corticosteroids. Short-term mindfulness training during these brief periods of hospitalization may represent an effective complementary therapy that might improve symptoms of depression, fatigue and cognitive symptoms.

Based on the reasoning outlined above, the current study had two purposes: First, the primary goal of the current work was to explore the feasibility and potential beneficial effect of short-term mindfulness training on depressive symptoms, fatigue, rumination, and cognition in PwMS in an acute-care hospital setting. To address this issue, a protocol for a short-term mindfulness intervention was developed and administered to participants during the routine clinical process. Since, to the best of our knowledge, the current study is the first in which such a short-term mindfulness training was implemented in an acute care hospital setting in PwMS and was exploratory in nature, no previous information on the validity and reliability of this short-term protocol was available upon study initiation. However, it should be noted that training sessions were particularly based on established work by Segal et al. [17], see Table 1 for details. At pre and post treatment assessments, depressive symptoms, fatigue, rumination, cognitive status, as well as trait mindfulness were examined. It was assumed that participation in the training would be associated with increased trait mindfulness. Further, it was expected that even in context of such a short-term treatment, depressive symptoms, fatigue, and rumination would be improved. Based on the assumption that a potential beneficial effect of the training would result in consistent alterations in these parameters, it was expected that increased trait mindfulness would be associated with decreased depression, fatigue, rumination and improved cognitive functioning following the intervention.

**Table 1:** Description of mindfulness training sessions

	Session	Description
1	Introduction	Presentation of the concept of mindfulness. Introduction of breathing exercise. Breathing exercises were used throughout the following sessions.
2	Bodily sensations	The body scan was performed. Participants were instructed to move their attentional focus through their body while retaining a mindful attitude.
3	Thoughts	In context of a breathing exercise, participants were encouraged to pay attention to upcoming thoughts without getting engaged in their content. Metaphors were introduced, e.g. imagining being on a beach and writing letters in the sand, that would be washed away by water afterwards. Participants were encouraged to adopt a neutral observing perspective, to step back from their current stream of thoughts and to experience that thoughts can be handled like neutral objects.
4	Emotions	The concept of radical acceptance of feelings was presented. The goal was to shape a mindful observant attitude to experience one’s own emotion irrespective of its consequences. Participants were invited to close their eyes and observe the thoughts and feelings in the present moment while retaining a mindful attitude.
5	Optional session	The first optional session covered the topic of s*elf-care and self-compassion*. Participants were encouraged to apply a mindful attitude of non-judgmental, curious, and gentle observation, acceptance and compassion to themselves and their needs.
6	Optional session	Applicability of mindfulness to daily activities, e.g. mindful eating, or brushing teeth mindfully. In the last session, participants could also pick exercises from their favorite session, from which they subjectively benefitted the most.

Note. The training consisted of four basic sessions (sessions 1–4) and two optional sessions (sessions 5–6). It was anticipated that during their relatively short stay in the acute-care hospital, the majority of patients would be able to participate in at least four mindfulness training sessions. As the duration in the hospital was known to vary considerably across patients, two optional sessions were offered for patients with a longer stay in the hospital

The second goal of the current work was to explore whether depression may indeed function as a mediator of the negative relationship between trait mindfulness and fatigue, building on previous results reported by Sauder et al. [35]. To this end, it was tested whether the cross-sectional results previously reported by Sauder et al. [35] could be replicated at pre- and post-treatment assessments, respectively, as well as for alterations that occurred across assessment points. A successful replication of these previous findings may provide further information on the working mechanisms of mindfulness training in reducing fatigue symptoms. As previously suggested, this finding may also have important clinical implications. According to Sauder et al. [35], fatigue might be regarded as disease-immanent, with a relatively close relation to inflammatory activity in PwMS [37, 38]. Depression on the other hand may additionally be attributable to adaptation difficulties that arise when dealing with the psychosocial consequences of MS. Based on this reasoning, depressive symptoms may be prone to be positively affected by psychotherapeutic interventions such as mindfulness training, whereas particularly disease-immanent default fatigue symptoms ought to be embraced with an accepting attitude during mindfulness training.

## Methods

### Participants and procedure

The current study was approved by the ethics committee of the University of Bamberg, Germany. Participants were recruited sequentially in the Department of Neurology, Klinikum Bayreuth GmbH, Germany, during the routine clinical process. Patients were admitted to the hospital for monitoring purposes, treatment optimization and treatment with immune-suppressive corticosteroids. In this context, the routine process involves staging, assessment of clinical parameters such as ambulation and cognition, as well as a critical evaluation of medication efficacy and functional therapies. Patients who reported symptoms of fatigue or depression during the routine exploration of the psychopathological status were offered to participate in the study. Additionally, the following inclusion criteria were applied: A confirmed diagnosis of MS based on revised McDonald criteria [39], a stay in the hospital of at least five days and an age of at least 18 years. All participants provided written informed consent before study entry. In sum, 34 patients were recruited.

**Fig. 1:**
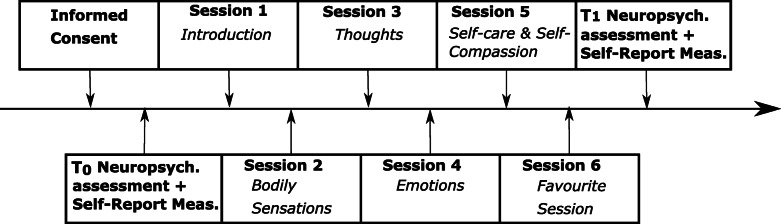
Study procedure. The mindfulness training consisted of four basic training sessions (Sessions 1–4) each with a different topic. It was assumed that the majority of patients would be able to complete these four sessions during their stay. In addition, two optional sessions were offered to patients, who stayed in the hospital for a longer period (Sessions 5–6)

An overview of the study procedure, respective assessment points (T_0_ = pre-treatment assessment, T_1_ = post-treatment assessment) and mindfulness sessions, including brief information about the content of the training exercises is provided in Fig. 1 (see also: Table 1 and section *mindfulness training*).

### Self-report measures

Depressive symptoms were assessed with the German version of the Center for Epidemiological Studies Depression Scale (CES-D [40, 41]). The CES-D examines depressive symptoms in the last week and contains 20 items, ranked on a scale from zero to three. A sum score (0–60), whereby a higher score reflects higher depressive symptoms, is derived. The German version of the CES-D provides a cut-off score of ≥24 as an indication of a clinically relevant depressive syndrome.

To assess the level of subjective fatigue symptoms, in line with previous work [35], the Wuerzburger Fatigue Inventory for Multiple Sclerosis (WEIMuS [42]) was administered. The WEIMuS contains 17 items, ranked on a scale from zero to four, yielding sum scores for its two subscales physical fatigue (range: 0–36) and cognitive fatigue symptoms (range: 0–32), as well as for general fatigue symptoms (0–68). Higher scores reflect higher subjective fatigue. Cut-off scores for clinically relevant physical (≥16), cognitive (≥17) and general fatigue (≥32) were implemented in line with previous work [35, 43]. The WEIMuS was previously reported to show high convergent validity with other measures of fatigue, particularly physical fatigue, as objectified in standardized walking tasks [44]. Moreover, it has been shown to be sensitive to alterations in fatigue induced by means of combined mindfulness and physical training exercises, as incorporated in Tai Chi training [19]. As such, it may be assumed to provide valid information on fatigue in intervention studies in PwMS.

The German short version of the Response Styles Questionnaire (RSQ [45]), based on the Response Styles Theory (RST [46, 47]), assesses rumination and distraction tendencies during negative mood. The RST postulates that rumination, i.e., excessive recurrent thinking about symptoms and negative self-aspects, yields an amplification of depressive symptoms, whereas distraction from ruminative behavior may lead to a reduction of depressive symptoms [47]. In this context, the RSQ compromises a total of 23 items across the three subscales of symptom-focused rumination (RSQ-SYM, eight items), self-focused rumination (RSQ-SELF, seven items) and distraction tendencies (RSQ-DIS, eight items). Items are rated on a scale from 1 to 4, respectively. Individuals with a high score on the subscale RSQ-SYM tend to ruminate about the causes and consequences of depressive symptoms, while those with a high score on RSQ-SELF ruminate about negative self-aspects. Individuals with a high score on RSQ-DIS on the other hand tend to distract themselves in a way of behaving and thinking, shifting their attention to other aspects of life. Hence, in this context higher distraction tendencies are to be regarded as beneficial. Sum scores were obtained for the subscales (ranges: RSQ-SYM, 1–32; RSQ-SELF, 1–28; RSQ-DIS, 1–32). The RSQ does not involve specific clinical cut-offs. However, the authors provide norm-values from a healthy comparison group and corresponding percentile ranks, that could be considered in the analysis (see section: statistical analysis for details). To be able to evaluate the clinical relevance of anticipated shifts in rumination from pre- to post-treatment assessments, individual scores reflecting a percentile rank > 80 (z-score = 0.84) relative to the norm values of the healthy comparison group were considered as suggesting elevated rumination of potential clinical relevance.

To measure the level of trait mindfulness, the German version of the Freiburg Mindfulness Inventory (FMI [48]) was implemented. The FMI contains 14 items, ranked on a scale from one to four yielding a sum score (0–56), with a higher score indicating a higher level of trait mindfulness. The items reflect the basic components of trait mindfulness, i.e., presence and acceptance.

### Neuropsychological assessment

The Symbol-Digit-Modalities-Test (SDMT [49]) was administered to assess information processing speed and attention. The SDMT is a common neuropsychological test for PwMS and recognized for its high sensitivity and specificity in detecting MS-related cognitive deficits [3, 50]. Participants were instructed to verbally pair as many symbols and digits as possible based on a fixed pattern within a time limit of 90 s.

To measure attention, the standardized and computerized Test of Attention Performance (TAP [8, 51]) with the subtest *Alertness* was performed. Participants were instructed to press a button as quickly as possible whenever a white cross in the center of a black screen appeared (intrinsic alertness). In a second condition, a probe warning tone preceded the appearance of the cross (phasic alertness). For the analyses, the median response times for intrinsic and phasic alertness were obtained.

### Mindfulness training

Sessions took approximately 45 min and took place in an individual setting. The training was administered by two psychologists, who administered mindfulness training on a regular basis in the clinical routine. An individual therapeutic setting was chosen over group therapy in context of the acute care hospital setting. Because of the high frequency of medical examinations during the medical monitoring and treatment process, it proved difficult to schedule a fixed time point for a group therapy. Routine treatment hence interfered with the simultaneous participation of a high number of patients at a regular time during the day.

The training was based on the MBCT manual by Segal et al. [17]. It was adjusted and shortened to four standard sessions and two optional sessions to account for the short amount of time available. Two optional sessions were implemented since the time spent in the hospital varied across patients. Participants who stayed longer than one week had the possibility to complete two optional sessions. After each session, participants were encouraged to engage in mindfulness practice during activities of everyday life. To this end, participants received handouts to write down what kind of practice was performed and how they experienced it. A short description of the sessions is depicted in Table 1 and Fig. 1.

### Statistical analyses

#### Pre- to post treatment alterations

To gain information about potential beneficial effects of mindfulness training, in a first step, multivariate repeated measures analyses of variance (MANOVA) were implemented as omnibus tests for two domains of variables. All models included the within-subjects factor TIME with two levels (pre vs. post therapy). In the first domain, the MANOVA included the self-report data, i.e., mindfulness, depression, general fatigue, cognitive fatigue, physical fatigue, self-focused rumination, symptom-focused rumination and distraction tendencies. In the second domain, the MANOVA included parameters of the cognitive tests, i.e., information processing speed (SDMT), and response times on the TAP tasks (tonic and phasic alertness). MANOVA were followed up by respective pre-post comparisons for each parameter, implemented by paired-samples t-tests. A Bonferroni correction was applied, that yielded a significance threshold of *p* = .0125 (i.e., *p* = .05/4; mindfulness, depression, fatigue, rumination) in the domain of the self-report measures. In the correction procedure, the number of domains was considered, as in the two measures that involved subscales, i.e., fatigue and rumination, the respective subscales showed highly significant intercorrelations (range of *r* = .47–.93). By the same rationale, a significance threshold of *p* = .025 was set in the cognitive domain (i.e., *p* = .05/2; SDMT score and response times on TAP alertness and TAP phasic alertness tasks, correlation between TAP alertness and phasic alertness *r* = .83). Hence, despite the exploratory nature of the current study, a conservative correction procedure was applied.

In order to provide further information on the clinical relevance of the anticipated alterations, for measures of fatigue and depression, the proportion of patients with scores above/below the respective clinical thresholds published in the original manuals was derived for pre- and post-treatment assessments. As noted above (section: self-report measures), the original manual of the rumination measure (RSQ), does not provide clinical cut-offs. However, individual percentile ranks relative to norm-values from healthy controls, as provided by the authors, were considered to be able to evaluate the clinical relevance of anticipated shifts in rumination from pre- to post-treatment assessments. To this end, an individual score reflecting a percentile rank > 80 (z-score = 0.84) relative to the healthy norm values was set as a threshold and respective proportions of patients above/below this percentile rank were derived for pre- and post-treatment assessments. Strictly speaking, a percentile rank > 84, reflecting a z-score of z = 1.036, indicative of an individual value exceeding one standard deviation of the norm values might be argued to be more specific. However, as the authors of the RSQ provide percentile ranks only in steps of 10, a rank of > 80 was chosen.

#### Consistency of alterations

In order to gain information about the consistency of the anticipated alterations, change scores were computed for each parameter, for which a significant shift from pre training (T_0_) to post training assessments (T_1_) was observable (e.g., mindfulessT_1_ – mindfulnessT_0_). In a descriptive analysis, the derived change scores were subsequently correlated by means of Pearson correlations to estimate the portion of congruent variance across assessment points.

#### Mediation analyses

**Fig. 2:**
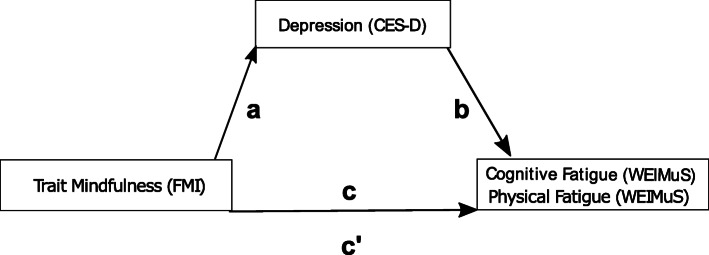
Schematic outline of the assumed mediation model; path a = regression analysis with depression used as criterion variable and trait mindfulness as predictor, path b = regression analysis with fatigue (i.e., cognitive, physical) as criterion variable and trait mindfulness and depression as predictors; path c = total effect of the model with trait mindfulness as predictor and fatigue (i.e., cognitive, physical) as criterion variable; c’ = direct effect (effect of trait mindfulness on fatigue, i.e., cognitive, physical, while controlling for depression ); the product of path a and b is defined as the indirect effect *ab*

It was tested whether trait mindfulness was negatively associated with cognitive and physical fatigue and whether the respective association was mediated by depression. To test if results reported by Sauder et al. [35] could be replicated in the current sample on a trait level, the indicated mediation analysis was implemented for self-report data obtained prior to the training and for data obtained following the training, separately for physical and cognitive fatigue, respectively. The same analysis was then implemented for the derived change scores. Respective mediation analyses were implemented by means of the PROCESS macro (version 3.5) by Hayes [52] using SPSS (version 27). With the PROCESS macro, regression coefficients and bootstrap confidence intervals of total, direct and indirect effects may be calculated. Originally, Baron and Kenny [53] outlined four steps required for mediation (for details see: [53] and [35]). More recently, it has been argued that not all four prerequisites have to be fulfilled and that the basic requirement of a mediation is met, if the indirect effect *ab* is significant [54, 55]. In the current work, this involved the path between mindfulness and depression (path *a*) and the path between depression and fatigue (path *b*). Therefore, for each mediation model, analyses focused on testing the significance of the indirect effect *ab*. In the analyses, bootstrapping with 5000 iterations to estimate the indirect effect was applied. A schematic outline of the assumed mediation model is depicted in Fig. 2.

## Results

### Datasets included in the final analyses

Out of the 34 participants who were recruited, four were excluded from the final analyses, as two were discharged from the hospital earlier than expected and two were not available at the appointed times due to other unforeseen medical examinations. Hence, for the final analyses, datasets of 30 participants remained for self-report measures of mindfulness, depression and fatigue. With respect to rumination, 29 datasets were available, since one participant did not complete the entire self-report measure at the first assessment. Because of time restrictions during their stay, two further participants did not complete the neuropsychological assessment. Clinical and demographic descriptive information as well as the number of sessions in which patients participated are provided in Table 2. The median duration that patients spent in the hospital was eight days with a median of four mindfulness training sessions during their stay. Three participants completed only three of the four basic sessions due to unforeseen medical examinations. Two patients with relatively longer stays in the hospital were exceptionally granted the possibility to participate in a total of eight and nine training sessions, respectively, in which case the “favorite session” was implemented on the respective additional occasions. In these two cases, training sessions were extended beyond the two optional sessions after a thorough ethical evaluation of their wish to continue practicing due to perceived benefits. As the current study was in essence exploratory in nature, the tradeoff between a rigidity of the study design on the one hand and the possibility to support patients with desired therapy on the other hand, was regarded as in favor of patients’ needs in these two exceptional cases. All cases were included in the primary MANOVA and follow-up analysis, however datasets of the two participants whose training exceeded six sessions (four basic, two optional) were removed in a secondary analysis, to rule out the possibility that the extra training may have confounded the results.

**Table 2:** *Sample description*

Demographic data	Statistic
N (male/female)	13/17
Age (*M*, *SD*)	53.3, 13.17
**Clinical characteristics**	
MS type (N: RRMS, SPMS, PPMS)	11, 18, 1
Treatment (N: current corticosteroid: yes/no)	23/7
Disability level (EDSS: median, range)	5.25, 2–8.5
Current relapse (N: yes/no)	4/26
Depression (N: yes/no)*	8/22
Cognitive Fatigue (N: yes/no)*	16/14
Physical Fatigue (N: yes/no)*	21/9
Days spent in hospital: median (range)	8 (4–21)
**Mindfulness intervention**	
Sessions completed: median (range)	4 (3–9)
Optional sessions completed: N (yes/no)	11/19

EDSS = Expanded Disability Status Scale; *M* = mean; PPMS = primary progressive MS; RRMS = relapsing remitting MS; *SD* = standard deviation; SPMS = secondary progressive MS; *presence of clinically relevant depressive symptoms and fatigue based on respective cut-off scores of the CES-D and WEIMuS, see self-report measures for details

### Pre- to post-treatment alterations: self-report measures

**Fig. 3:**
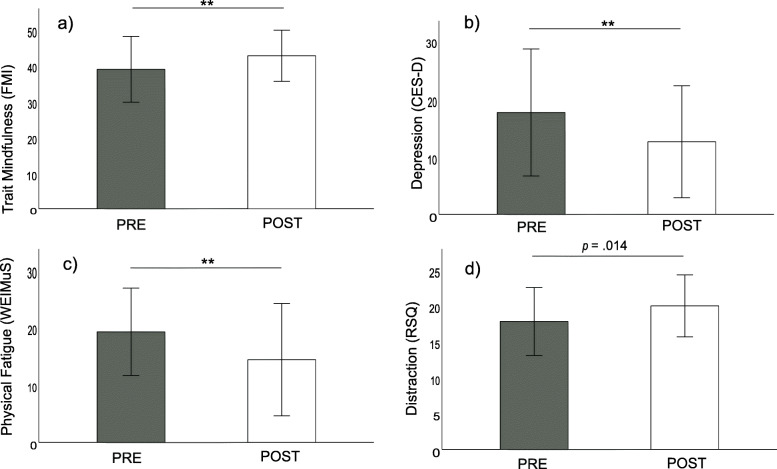
Mean scores on outcome parameters across assessments prior to (PRE) and following the mindfulness training (POST), displayed for trait mindfulness (a), depression (b) and physical fatigue (c), with respective *p*-values below the Bonferroni-corrected threshold of *p* <.0125. Note that all changes remained significant when the N = 2 cases whose training exceeded six sessions were removed. In case of distraction tendencies (d), there was a trend for an improved adaptive ability to steer attention away from arising negative mood (p = .014). Error bar represent +/- 1 standard deviation; CES-D = Center for Epidemiological Studies Depression Scale; FMI = Freiburg Mindfulness Inventory; RSQ = Response Styles Questionnaire; WEIMuS = Wuerzburger Fatigue Inventory for Multiple Sclerosis; ** = significant at *p*< .01

The omnibus MANOVA yielded a significant main effect of TIME *F*(7, 22) = 2.797, *p* = .028, *η*_*p*_^*2*^ = .516. Results of the follow-up paired-samples t-tests are reported in Table 3 and displayed graphically in Fig. 3. As displayed, a significant increase in trait mindfulness was observed, as well as a significant decrease in depressive symptoms. Moreover, general fatigue and physical fatigue were significantly decreased following the training. When the datasets of the two participants whose training exceeded six sessions were removed, alterations in all indicated parameters remained significant, with respective *p*-values remaining below the Bonferroni-corrected significance threshold. Overall, the indicated alterations were characterized by moderate effect-sizes (range of Cohen’ *d =* 0.50–0.73).

**Table 3:** Pre- to post-treatment changes on self-report measures

	pre		post		sig.	effect size
	*M*	*SD*	*M*	*SD*	*p*	*d*
Mindfulness (FMI)	38.68	9.12	42.47	7.10	**.003**	0.50
Depression (CES-D)	17.55	10.94	12.50	9.66	**.002**	0.54
General Fatigue (WEIMuS)	33.27	14.42	26.03	18.62	**.006**	0.58
Physical Fatigue (WEIMuS)	19.07	7.52	14.27	9.67	**.001**	0.73
Cognitive Fatigue (WEIMuS)	14.20	8.19	11.93	9.62	.070	0.31
Self-focused rumination (RSQ)	13.02	4.65	12.28	3.38	.206	0.14
Symptom-focused rumination (RSQ)	16.90	5.29	15.86	5.01	.149	0.19
Distraction (RSQ)	17.72	4.71	19.90	4.29	.014	0.41

Note. Pre- to post-treatment alterations examined by means of paired-samples t-tests. *p*-values below the Bonferroni-corrected threshold of *p* = .0125, i.e., corrected for each self-report domain (mindfulness, depression, fatigue, rumination), displayed in bold (one-tailed). Mean values in general fatigue and physical fatigue were above respective clinical cut-offs (≥32 and ≥ 16) at pre-treatment assessments and below cut-offs at post-treatment assessments. See results section for a detailed description of the proportion of patients who shifted from clinically elevated to sub-clinical levels. A trend for an improved adaptive ability to engage in distractive behavior when confronted with negative mood was observed post-treatment. CES-D = Center for Epidemiological Studies Depression Scale; *d* = Cohen’s *d* for repeated measures; FMI = Freiburg Mindfulness Inventory, M = Mean, RSQ = Response Styles Questionnaire, SD = Standard Deviation, WEIMuS = Wuerzburger Fatigue Inventory for Multiple Sclerosis

With regards to the clinical relevance of the observed alterations in fatigue, prior to the intervention, 60% (*N* = 18) of the participants scored above the clinical threshold of general fatigue. Following the intervention, this rate was reduced to 43% (*N* = 13). A corresponding pattern was observed for both physical fatigue (pre: 70%, *N* = 21; post: 53%, *N* = 16) and cognitive fatigue (pre: 53%, N = 16; post: 33%, *N* = 10). Across fatigue scales, the proportion of patients whose symptoms improved from a clinically relevant manifestation to a subclinical level hence ranged from 17 to 20%. A similar pattern was observed for depression, where a 10% decrease of patients who displayed clinically relevant depressive symptoms was observable (pre: 27%, *N* = 8; post: 17%, *N* = 5).

There also was a marginally significant increase in distraction tendencies characterized by a small to medium-sized effect (*d =* 0.41, Table 3). This suggests that following the training, patients tended to perceive an improved ability to steer their attention away from negative mood. Symptom-focused and self-focused rumination remained relatively stable. Exploratory analyses revealed that prior to the intervention, 24% (*N* = 7) patients showed clinically elevated symptom-focused rumination, with a minor reduction to 20% (*N* = 6) following the mindfulness training. The same pattern was observed for self-focused rumination (pre: 14%, *N* = 4; post: 7%, *N* = 2) and a corresponding reversed pattern for adaptive distraction tendencies (pre: 10%, *N* = 3; post: 14%, N = 4).

### Cognitive measures

In case of the cognitive measures, the MANOVA did not show a significant main effect of TIME *F*(3, 25) = 0.240, *p* = .867, *η*_*p*_^*2*^ = .028. Paired-samples t-tests were insignificant for each variable, i.e., SDMT, TAP alertness and TAP phasic alertness (all *p*-values > .05).

### Descriptive inter-correlations of change scores across assessments

**Fig. 4:**
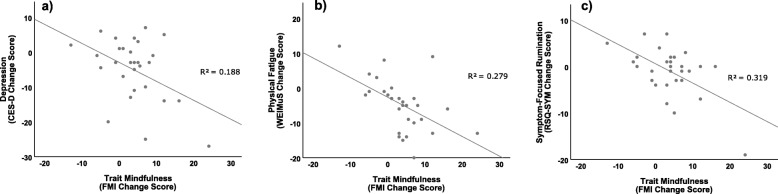
Significant correlations between the change score of trait mindfulness (FMI) and depression (CES-D; a), physical fatigue (WEIMuS, b), and symptom-focused rumination (RSQ, c); CES-D = Center for Epidemiological Studies Depression Scale; FMI = Freiburg Mindfulness Inventory; WEIMuS = Wuerzburger Fatigue Inventory for Multiple Sclerosis; RSQ = Response Styles Questionnaire; R^2^ = R-squared (coefficient of determination)

Significant negative correlations between change scores of mindfulness and depression, *r*(30) = − .434, *p* = .017 (Fig. 4a), and between mindfulness and physical fatigue were observed, *r*(30) = − .528, *p* = .003 (Fig. 4b), indicating that an increase in mindfulness was associated with decreased symptoms. Exploratory analyses reavealed that mindfulness was not associated with distraction tendencies *r*(30) = .227, *p* = .237. Furhter exploratory analyses considering the remaining rumination subscales revealed that an increase in mindfulness was associated with a decrease in symptom-focused rumination *r*(30) = − .565, *p* = .001 (Fig. 4c), but not with changes in self-focused rumination *r*(30) = − .125, *p* = .518.

### Mediation analyses

**Fig. 5:**
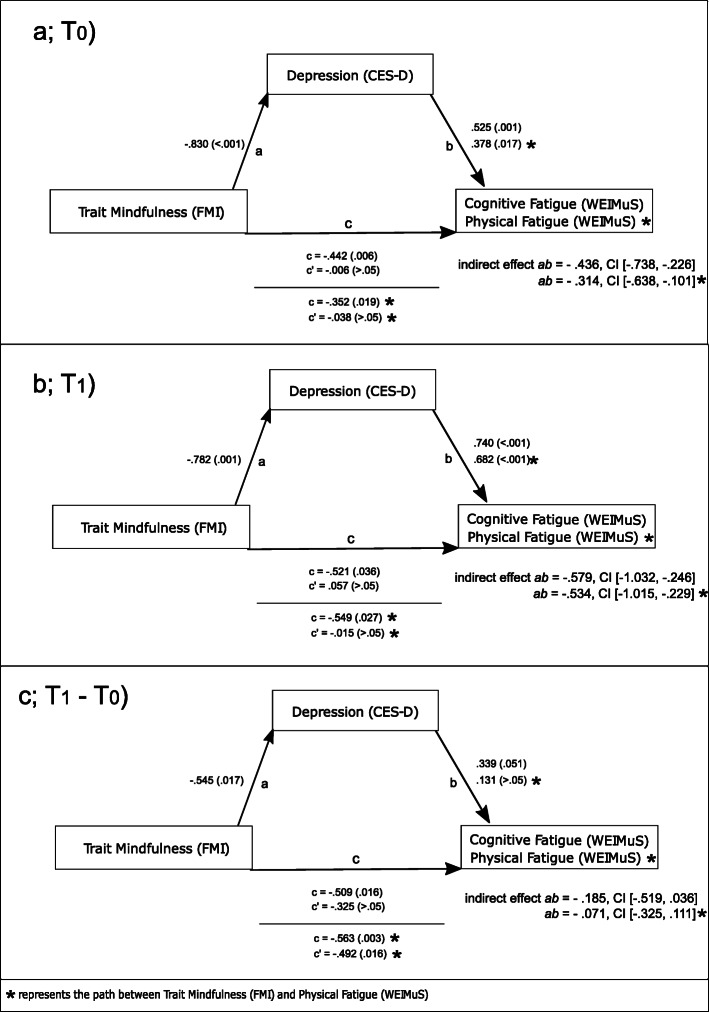
Outline of the results of the mediation analyses at T_0_ (a)_1_ T_1_ (b) and for the concurrent change scores (T_1_-T_0_, c). Results of the regression analyses are depicted as unstandardized B coefficients with respective *p*-values in parenthesis for each path (path c = total effect of trait mindfulness on fatigue , path c’ = direct effect while controlling for depression); CI = 95% Confidence Interval

*T*_*0*_. As displayed in Fig. 5a, in the cross-sectional mediation analysis that focused on self-report measures obtained prior to the intervention (*T*_*0*_), a significant total effect of trait mindfulness on cognitive fatigue was observed (unstandardized B = −.442, *p* = .006). When depression was entered as a potential mediator, trait mindfulness predicted depression (B = −.830, *p* < .001), depression in turn predicted cognitive fatigue (B = .525, *p* = .001) and the previously significant effect of trait mindfulness on cognitive fatigue became insignificant (B = −.006, *p* > .05). In this context, the association between trait mindfulness and cognitive fatigue was significantly mediated by depression, indirect effect *ab* = −.436, 95% Confidence Interval (CI) [−.738, −.226]. As depicted in Fig. 5a, the same analysis was also implemented for physical fatigue. In this case, the relationship between trait mindfulness and physical fatigue was also significantly mediated by depression, indirect effect *ab* = −.314, 95% CI [−.638, −.101].

**Fig. 6:**
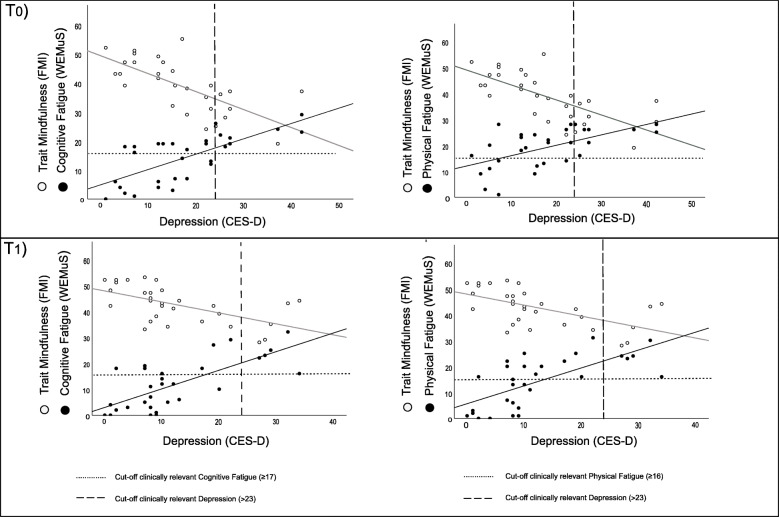
Scatterplots of the correlation between trait mindfulness, fatigue (i.e., cognitive, physical fatigue) and depression with dashed and dotted lines illustrating the cut-off scores for clinically relevant fatigue and depression before (T_0_) and after training (T_1_); CES-D = Center for Epidemiological Studies Depression Scale, FMI = Freiburg Mindfulness Inventory; WEIMuS = Wuerzburger Fatigue Inventory for Multiple Sclerosis

*T*_*1*_. In the second cross-sectional mediation analysis that focused on self-report measures obtained following the intervention (*T*_*1*_), the same analysis was implemented (see Fig. 5b for details). Also in this case, depression functioned as a significant mediator of the relationship between trait mindfulness and cognitive fatigue (*ab* = −.579, 95% CI [− 1.032, −.246]) as well as of the relationship between trait mindfulness and physical fatigue (*ab* = −.534, 95% CI [− 1.015, −.229]). Scatterplots illustrating the association between depression on the one hand, as well as mindfulness and fatigue on the other hand at *T*_*0*_ and *T*_*1*_ are also depicted in Fig. 6.

*Change scores T*_*1*_*-T*_*0*_. As depicted in Fig. 5c, in case of the change scores obtained across pre and post assessments, a significant total effect of trait mindfulness on fatigue was observed (total effect on cognitive fatigue: B = −.509, *p* = .016; physical fatigue: B = −.563, *p* = .003). When depression was controlled, respective coefficients reflecting the direct effect of mindfulness on fatigue ceased to be significant for cognitive fatigue, albeit this was not the case for physical fatigue. The mediating (indirect) effect of depression did not reach significance, respectively (cognitive fatigue, *ab* = −.185, 95% CI [−.519, .036]; physical fatigue: *ab* = −.071, 95% CI [−.325, .111].

## Discussion

The primary goal of the current study was to explore the feasibility and potential beneficial effects of short-term mindfulness training on psychological symptoms (fatigue, rumination, depression) and cognitive functioning in PwMS during a brief period of hospitalization. While the design of the current study was exploratory in nature and definite conclusions concerning cause and effect relationships cannot be derived, overall results provide preliminary support for potential benefits of the short-term training.

### Alterations in mindfulness, depression, fatigue, and rumination

In particular, self-reported trait mindfulness was significantly increased during training, while depressive symptoms and fatigue were significantly decreased. Moreover, descriptive inter-correlations of change scores of mindfulness on the one hand and depression and fatigue on the other hand, were highly significant. This implies that participants who exhibited higher trait mindfulness after the intervention, displayed improved symptoms of depression and fatigue. As indicated, the design of the current study was exploratory and as there was no comparison group, effects per se were not addressed. Nevertheless, it is noteworthy that all indicated parameters were altered in line with the original hypotheses, compatible with a potential benefit due to the training. This suggests a coherent pattern of the current results in line with the concurrent literature [26–29] and implies that PwMS may profit from mindfulness training even during brief periods of hospitalization.

In the domain of fatigue, physical fatigue, but not cognitive fatigue decreased following the training. This significant alteration in physical fatigue might be explainable by the nature of the training applied. Certain mindfulness techniques were based on physical sensations (e.g., body scan) to make the idea behind mindfulness more tangible. We suggest that our training, that involved the development of awareness towards physical sensations, may have enabled patients to adopt a mindful meta-perspective of the physical signs of fatigue. Being aware of exhaustion, and thereby allowing an early detection of signs of fatigue, might also prompt patients to engage in recreational behavior and rest, when necessary.

The reason for the lack of a significant decrease in cognitive fatigue remains somewhat unclear. In recent studies, where fatigue was examined in relation to mindfulness training [26, 27], the authors used a different self-report measure than in the current study to quantify fatigue-related symptoms (Modified Fatigue Impact Scale, MFIS [56]). The MFIS contains a physical, cognitive and a psychosocial subscale. As previous work predominantly focused on the global score derived from these subscales, it remains unclear which of the subscales (physical, cognitive, psychosocial) particularly contributed to the reported association. Therefore, the question as to whether mindfulness training in general might have a differential impact on physical vs. cognitive fatigue remains to be addressed in more detail. It is noteworthy however, that on the clinical scales included in the current work, i.e., scales of fatigue and depression, the proportion of patients whose symptoms improved from a clinical to a subclinical level increased consistently from pre- to post-treatment assessments. The fact that 17–20% of the treated patients improved to a subclinical fatigue level and that 10% improved to a subclinical level of depression suggests that that the observed improvements bear clinical relevance.

Concerning rumination behavior, in the current study, there was only a trend for scores on the distraction subscale to be increased following the intervention. It should be noted however that patients did not report distraction in the common sense of the word (i.e., inattention) following the intervention. In essence, the distraction subscale reflects in how far individuals are able to attend to neutral or positive alternatives during arising negative mood. According to the Response Styles Theory [46, 47], distraction tendencies are associated with less depressed mood. In this context, the potential benefit of mindfulness training to improve patients’ skills to distract themselves from negative mood, e.g., by focusing on their breath, has previously also been demonstrated on a neurophysiological level [57]. Consequently, it may be speculated that also in the current work, mindfulness training tended to enable patients to steer their attention away from arising negative mood states. Compatible with this reasoning, change scores between trait mindfulness and symptom-focused rumination revealed that an increase in trait mindfulness was associated with a decrease in symptom-focused rumination. Thus, participants who became more mindful following the intervention also tended to ruminate less about the causes and consequences of their depressive symptoms. Nevertheless, as particularly the increase in the perceived ability of patients to distract themselves from arising negative mood was only observable on a trend level, the reasoning above remains somewhat speculative and ought to be addressed by future work in more detail.

### Mediating role of depression in the reduction of fatigue by mindfulness training

In the cross-sectional analyses, depression mediated the relationship between trait mindfulness and cognitive/physical fatigue at both pre- and post-treatment assessment. These results replicate findings previously reported by Sauder et al. [35] where the same mediation effect was observed in a cross-sectional study. As these previous findings could be replicated in a new independent sample and consistently across two assessment points in the current work, the mediating role of depression in the antagonistic relationship between trait mindfulness and fatigue may be regarded as robust in cross-sectional examinations. It has been suggested that fatigue symptoms may be considered as relatively closely related to inflammatory activity, especially increased cytokine levels [37, 38] in PwMS, i.e., disease-immanent, whereas depression is additionally affected by adaptation difficulties to the psychosocial consequences of MS. Hence, one may expect depression to be more prone than fatigue to be beneficially affected by mindfulness practice. The current results are generally compatible with this reasoning since the negative relation between trait mindfulness and fatigue was attributable to the mediating effect of depression.

While the current work provides strong support for depression as a mediator between trait mindfulness and fatigue in cross-sectional analyses, in the longitudinal analyses across assessment points, mixed results were obtained. A significant total effect of altered mindfulness on altered fatigue was observable and this effect was reduced when depression was controlled. Nevertheless, depression did not emerge as a significant mediator in this constellation, disconfirming the hypothesis that the cross-sectional mediating role of depression may generalize to an interventional setting. There are several reasons that may account for this finding: First, it should be noted that the sample size of the current study was relatively small and given the minor trend of a mediating effect (i.e., reduction of the total effect of altered mindfulness on cognitive fatigue when depression was controlled), it cannot be ruled out that depression may have emerged as a significant mediator in a larger sample. Some previous studies that successfully addressed treatment mechanisms of MBI’s in PwMS have used similarly small samples (e.g., [58], *N* = 40). However, other successful work that has focused on mediation analyses was based on considerably larger data bases (e.g., [34]), *N* = 755). Given this heterogeneity of sample sizes in the literature and considering the replication of positive findings in the cross-sectional analyses of the current work, it may be suggested to repeat this specific analysis of the current work using a larger sample.

Additionally, it may be suggested that alternative self-report measures of trait mindfulness, depression and fatigue might be better suited to address mediation effects in longitudinal analyses. Based on the current results, it appears feasible to recommend that future studies address this issue in more detail.

### Cognitive performance

With regard to the cognitive measures, no significant changes were observed. The existing literature suggests that MBIs might improve cognitive function in PwMS [30, 31]. However, as noted in the introduction, examining potential changes on cognitive measures remains a methodological challenge [30]. Therefore, further studies are required to determine if MBIs represent an effective method to improve cognitive functions in PwMS.

### Practicability & Implementation

One important aspect of the current work concerns the feasibility of short-term mindfulness training and the question how practicable receiving mindfulness training in the acute care setting is. The authors of the current work assume that short-term and intensive mindfulness training can be effective in teaching the basic components and skills of mindfulness practice. To strengthen these skills, patients were encouraged to continue practicing at home and received further practice material about mindfulness, similar to MBSR [16] and MBCT [17]. Overall, patients apparently appreciated the training. This is supported by the high study retention and adherence to the treatment. In sum, 30 of 34 patients completed at least three sessions and the follow-up assessment, despite the involved short time interval. Further, the mindfulness training could be successfully implemented in the routine treatment plan of the acute care hospital setting.

### Limitations

Since the current study was exploratory in nature and did not involve a comparison group, the design does not allow definitive conclusions about the effects of the training. A potential confounding factor might be that a considerable number of participants received steroid therapy. Corticosteroid therapy can be accompanied by changes in mood and cognition [59]. However, as results obtained with the self-report measures were coherent and showed convergent validity, it appears unlikely that they were confounded by unspecific effects of corticosteroid treatment. Another important issue is that the current study did not include any measures on pain. It has recently been suggested that pain, fatigue and depression might represent a symptom cluster in PwMS [60]. Since pain has been shown to be associated with both depression [61] and fatigue [62], including pain measures in future work may prove particularly useful to examine the working mechanisms of MBIs in PwMS in more detail.

It should also be noted that the fatigue scale that was implemented in the current work, i.e., the German WEIMuS, does not represent a measure that is frequently used in internationally published studies. This may somewhat limit the generalizability of the current results and options to compare the current findings with those from future work. On the other hand, as noted in the methods section, work from our group has shown that the WEIMuS shows high convergent validity with other measures of fatigue, particularly objectively quantified physical fatigue [44]. It is also characterized by good convergent validity with other more established self-report measures of fatigue, such as the MFIS [63]. Further, it has also been used successfully in previous work on the effects of MBIs in PwMS and was shown to be a sensitive instrument [19]. Nevertheless, other measures such as the Fatigue Severity Scale (FSS [64];) or the MFIS would probably be feasible to be applied in future work for reasons of comparability.

Finally, the duration of the training was rather short with a median of four sessions. It may be speculated that particularly changes in cognitive functioning might only become evident after a long-term training. Moreover, future work should address the open question how long PwMS may profit from the participation in such a short-term MBI and whether they are inclined to keep practicing following their release from the hospital. To this end, further information from long-term follow-up examinations is required.

### Conclusion & Implications

The results of the current study suggest that short-term mindfulness training during a brief period of hospitalization may be beneficial for PwMS. The current work may therefore contribute to a better understanding and a further extension of the applications of MBIs. Implementing short-term mindfulness training in this clinical context might offer a complementary treatment method in the neuropsychological repertoire. Mindfulness training could be shown to be a feasible application in the hospital routine treatment plan. Based on these preliminary findings, future studies may address potential effects in more detail by using a randomized study design. Finally, the results extend the current state of knowledge by examining the mechanisms underlying the beneficial effect of mindfulness training on fatigue. Depressive symptoms may be prone to be positively affected by mindfulness training. Fatigue symptoms on the other hand may to a certain extent be regarded as default, i.e., disease-immanent and therefore relatively rigid in their response to behavioral interventions that have the goal of their substantial and sustained improvement. Results of the mediation analyses suggest that improved fatigue symptoms may be mediated by improvements in depression. MBIs in PwMS therefore ought to encourage patients to develop a mindful skillset that enables them to embrace particularly default fatigue symptoms with an accepting attitude.

## Data Availability

The data that support the findings of this study are available upon request from the corresponding author, PMK.
